# Co-ordinate-based positional embedding that captures resolution to enhance transformer’s performance in medical image analysis

**DOI:** 10.1038/s41598-024-59813-x

**Published:** 2024-04-23

**Authors:** Badhan Kumar Das, Gengyan Zhao, Saahil Islam, Thomas J. Re, Dorin Comaniciu, Eli Gibson, Andreas Maier

**Affiliations:** 1 Digital Technology and Innovation, Siemens Healthineers, Erlangen, Germany; 2 Pattern Recognition Lab, Department of Computer Science, Friedrich-Alexander-Universität Erlangen-Nürnberg, Erlangen, Germany; 3 Digital Technology and Innovation, Siemens Healthineers, Princeton, NJ USA

**Keywords:** Engineering, Biomedical engineering, Computer science

## Abstract

Vision transformers (ViTs) have revolutionized computer vision by employing self-attention instead of convolutional neural networks and demonstrated success due to their ability to capture global dependencies and remove spatial biases of locality. In medical imaging, where input data may differ in size and resolution, existing architectures require resampling or resizing during pre-processing, leading to potential spatial resolution loss and information degradation. This study proposes a co-ordinate-based embedding that encodes the geometry of medical images, capturing physical co-ordinate and resolution information without the need for resampling or resizing. The effectiveness of the proposed embedding is demonstrated through experiments with UNETR and SwinUNETR models for infarct segmentation on MRI dataset with AxTrace and AxADC contrasts. The dataset consists of 1142 training, 133 validation and 143 test subjects. Both models with the addition of co-ordinate based positional embedding achieved substantial improvements in mean Dice score by 6.5% and 7.6%. The proposed embedding showcased a statistically significant advantage p-value< 0.0001 over alternative approaches. In conclusion, the proposed co-ordinate-based pixel-wise positional embedding method offers a promising solution for Transformer-based models in medical image analysis. It effectively leverages physical co-ordinate information to enhance performance without compromising spatial resolution and provides a foundation for future advancements in positional embedding techniques for medical applications.

## Introduction

Vision transformers^1^ have emerged as a breakthrough in computer vision by introducing a paradigm shift from the convolutional neural networks to self-attention for image recognition tasks. Recent advancements in ViTs have yielded remarkable results and demonstrated their potential in various computer vision applications. The advantages of Vision Transformers lie in their ability to capture global dependencies in images by reducing the spatial inductive biases of locality.

Application of ViTs has expanded to medical imaging for tasks such as classification, segmentation, and detection^2–7^. Existing architectures require heavily constrained input characteristics such as a fixed input data size. However, medical imaging includes heterogeneous acquisition protocols, differing in size and resolution. Currently resampling or resizing methods are typically used for medical images in the pre-processing step to mitigate size differences. However, resampling involves changing the pixel dimension which can result in a loss of spatial resolution. This is particularly important in clinical applications where small or thin structures are critical. Resampling also involves interpolation which can cause information loss. For these reasons, Transformer models’ performance may be compromised when applied to medical images with heterogeneous acquisition protocols.

ViT-based architectures have been widely used in various medical imaging tasks such as detection of breast cancer in mammography images^2^, detection of lung nodule in computed tomography (CT) scans^3^ and achieved competitive performance compared to traditional convolutional neural network (CNN) architectures. For 3D medical image segmentation, UNETR^8^ was proposed which used a pure Transformer to learn sequence representations and performed very well on BTCV and MSD dataset^9^. Recently, another Transformer variant, Swin Transformer^10^ was introduced which is computationally very efficient due to the concept window-based-attention. SwinUNETR^11^, a combination of Swin Transformer encoder and UNET decoder was proposed for medical image segmentation, demonstrated remarkable performances in CT and MRI scans.

Positional embeddings play a crucial role in both Vision and Swin Transformers by providing position information of every patch, since self-attention itself is permutation-equivariant. There are several positional embedding techniques. The absolute positional encoding^12^ is the most frequently used in which fixed absolute values (represented by sine and cosine functions with varying frequency) are used as embeddings. In learnable positional embeddings^1^, embeddings are treated as model parameters and updated during the training process. All these positional embeddings methods are designed for natural images which usually don’t have physical co-ordinate information and these embeddings only incorporate patch-position information. For medical images, physical co-ordinates and pixel spacing are important for perfect alignment of multiple images.

Tomographic medical imaging devices, such as MRI and CT, have fixed physical co-ordinate systems linked to the physical structure of the device, storing each pixel’s position during acquisition. Each pixel’s position in the physical co-ordinate can be recovered from the metadata of that medical image. In this study, we propose “Co-ordinate based positional embedding” to capture the physical co-ordinate and resolution information from the metadata. Typically, heterogenous datasets can have medical images with different fields of view, resolutions, and matrix sizes in real-world image co-ordinates. We hypothesized that the physical-co-ordinate information along with resolution difference for each data sample can improve the performance of the Transformer models as it will help the neural network to understand the field of view, resolution, and matrix size differences of different input images. It will also allow us to use Transformer based architectures without resampling/resizing the input image and avoid information loss during preprocessing.

## Methods

This retrospective study was compliant with the health insurance portability and accountability act (HIPAA). The dataset was collected and anonymized from three different centers in USA and China; each hospital’s institutional review board approved this study for human research with waiver of informed consent. All methods were performed in accordance with the relevant guidelines and regulations.

We used a magnetic resonance imaging (MRI) dataset with 1142 training, 133 validation and 143 test subjects for acute/subacute brain infarct segmentation and for each subject two MRI contrasts were used: Axial apparent diffusion coefficient (AxADC) and Axial Trace (AxTrace). The images were acquired on scanners from Siemens Healthineers AG and, GE Medical Systems with echo times ranging from 66.0 ms to 131.8 ms, and repetition times ranging from 3200.0 ms to 17000 ms. Additionally, slice thickness varied for various patients’ acquisitions between 18 to 76 (details in supplementary materials). The dataset has a male to female patient ratio of 39:37 and includes scans from patients ranging in age from 18 years to over 80 years which makes it diverse.

The manual segmentation of acute and subacute infarct lesions was performed on AxTrace contrast image series by an expert radiologist (T.J.R.) from Siemens Healthineers. The radiologist used the medical image segmentation software ITK-SNAP version 3.8.0. The AxTrace image series and corresponding Apparent Diffusion Coefficient (ADC) image map were loaded into the software and reviewed by the radiologist. Areas, within the brain parenchyma, of hyperintensity in the TraceW image series with hypo or iso-intensity in the ADC map were considered positive for recent (acute to subacute) infarct by the radiologist and delineated as such in an image mask using the software tool. The radiologist identified at least one infarct lesion in each of the the data samples used in this study. Each subject may have different acquisition protocols, which may lead to different image resolution, origin, field of view and matrix size, but since for each subject both ADC and Trace are derived from the same diffusion weighted images, AxADC and AxTrace have the same sampling grid for each subject. As a part of pre-processing, we performed normalization and random cropping/resampling. For image normalization, we treated each channel individually. This involved computing the mean and standard deviation for each channel, followed by the subtraction of the mean from every pixel value, subsequently dividing by the computed standard deviation. Our random cropping involves extracting image regions with specific size regions of interest (ROIs). These regions can be cropped from random positions. We performed resampling to compare the performance with random cropping. Here both Trace and ADC images are resampled to a particular shape. We also performed data augmentation by random flipping the image in each direction with probability of 0.5.

**Figure 1:**
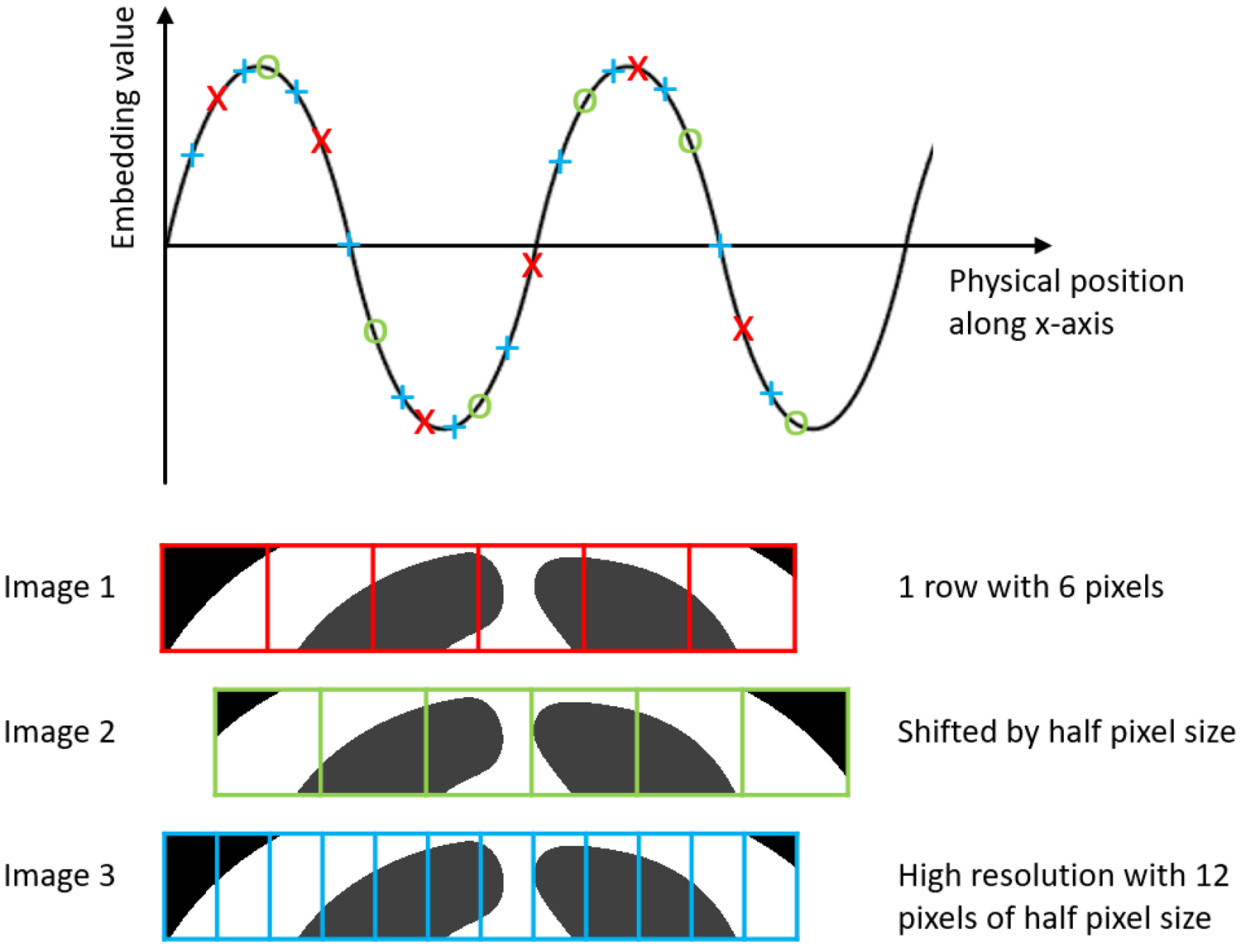
The mechanism of physical-coordinate-based positional embedding.

From metadata information: origin and pixel spacing, we can derive the physical coordinate of the center of each voxel. Then, we generate a 3D radial sinusoidal structure in the device’s physical coordinate system centered at the device’s coordinate system origin, whose value at each location is a function of the location’s coordinates in the device’s physical coordinate system as shown in the formula below:

1 $$\begin{aligned} \textit{PPE(x,y,z)} = \frac{{\sin (\omega \sqrt{x^2+y^2+z^2})}}{S} \end{aligned}$$

Here, *PPE* stands for proposed positional embedding where S, the scaling factor and $\omega$, the angular frequency are the hyperparameters. We scaled the proposed embedding to ensure positional similarity does not dominate the semantic similarity. Additionally, the manipulation of angular frequency serves as a means to modulate the sinusoidal structure, enabling its contraction or expansion as needed. The sinusoidal structure is being generated based on the Euclidean distance from the origin. The detailed computation of the proposed positional embedding is presented in Algorithm 1. This embedding provides physical-co-ordinate information along with resolution difference for different input images to the Transformer and it works in addition to the regular positional embedding. The PPE is a dense metric guidance at the pixel level as we added this to every pixel. However, given the periodicity of the sinusoidal function and the possibility of repeated values, the regular positional embedding aids in mitigating ambiguities. The regular embedding serves at the macro scale, while the PPE serves at the micro scale and is encoded to the features latent space. Hence, our PPE can add more feature-level useful information to the attention mechanism, and serves as a complement to the patch-wise regular embeddings. In conjunction with regular positional embeddings, the incorporation of our physical embedding strategy can fully harness the capabilities of the Transformer architecture. The mechanism of calculating the proposed positional embedding for images with different resolution, matrix size and shifts is shown in Fig. 1.

**Algorithm 1:**
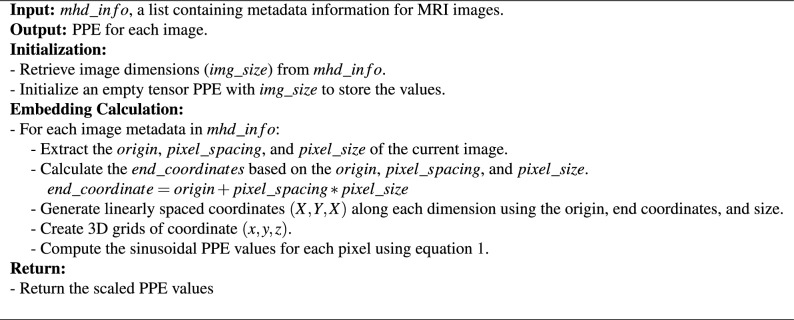
Computation of proposed positional embedding (PPE).

**Figure 2:**
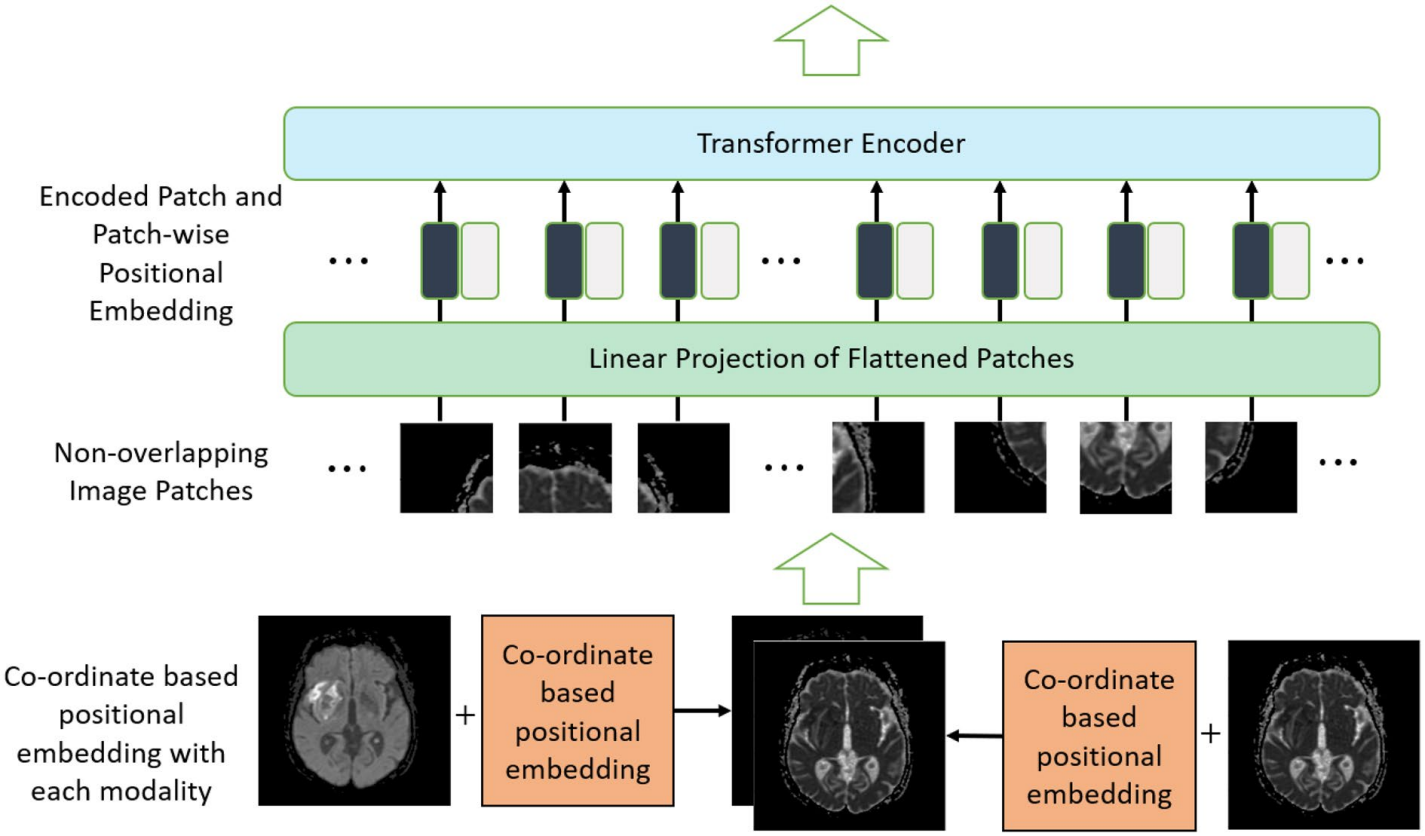
Vision transformer architecture with co-ordinate-based positional embedding. Proposed positional embedding is added to each modality separately followed by the creation of non-overlapping patches. Linear projection is used on the flattened patches and patch-wise positional embedding is added before sending these patches to the transformer encoder.

At first, we calculate the coordinate-based positional embedding for the input image. We then add this coordinate embedding information with the image $I_{\text {with\_PPE}} = I + PPE(x,y,z)$, here $I \in \mathbb {R}^{(H \times W \times D \times C)}$ represents the input image. Subsequently, we divide the new image $I_{\text {with\_PPE}}$ into non-overlapping patches denoted as $Patches \in \mathbb {R}^{(N \times (P^3 \cdot C))}$, where (*P*, *P*, *P*) signifies the resolution of each patch, and $N = \frac{(H \times W \times D)}{(P^3)}$ represents the length of the sequence.Then we perform linear projection on flattened patches and add patch-wise positional embedding $PE_{\text {patch-wise}} \in \mathbb {R}^{N \times K}$ with them to create input tokens of the Transformer, $Tokens = \text {LinearProjection}(Patches) + PE_{\text {patch-wise}}$, where $Tokens \in \mathbb {R}^{(N \times K)}$. Here, *K* represents the dimensionality of the token embeddings. This addition of patch-wise positional embedding with each token is similar to the original Vision Transformer^1^. Next, these tokens with both patch-wise and co-ordinate-based embedding are fed to the Transformer encoder. Detailed overview of the proposed embedding with Vision Transformer architecture is shown in Fig. 2.

We evaluated our co-ordinate-based positional embedding with two Commonly used baseline Transformer models: UNETR^8^ and SwinUNETR^11^ to demonstrate the effectiveness of this embedding on both Vision Transformer and Swin Transformer. While UNETR, based on ViT, performed exceptionally well in segmentation tasks with BTCV and MSD datasets, SwinUNETR, based on Swin Transformer, ranked among top performing approaches for the 2021 Brain Tumor Segmentation (BraTS) challenge. We compared the results of acute/subacute infarct segmentation with and without the addition of the proposed embedding for both these models.

We trained both UNETR and SwinUNETR models on the NVIDIA Tesla V100 SXM2 cluster for 300 epochs and saved the best validation accuracy model for performance evaluation. A batch size of 1 was employed during training due to the limited GPU memory caused by the large size of the model. This limitation arises from the extensive memory requirements of the Transformer’s processing of 3D data. The learning rates were identical (0.0001) for all the experiments during training. We performed our experiments with two different angular frequency 0.1 and 1. Dice similarity coefficient and 95th-percentile Hausdorff Distance (HD95) were used for quantitative evaluations on validation and test sets. Two-sided pairwise Wilcoxon signed rank test^13^ with Bonferroni correction was used to compare the Dice scores from different methods. The experiments were implemented using PyTorch(v1.12.1) and the Monai^14^(v1.1.0) framework.

In addition to the above infarct segmentation dataset, we also validated the performance of our proposed method using the BraTS 2021 dataset^15^ which includes 1000 training and 250 validation samples consisting of T1-weighted, T2-weighted, Flair, and T1-ce contrasts. The ground truth of the brain segmentation includes tumor core(TC), whole tumor(WT), and enhancing tumor(ET).

## Results

With the proposed embedding both UNETR and SwinUNETR model outperformed the other methods as shown in Table 1. The UNETR model, when combined with the proposed embedding, achieved a Dice score of 0.560. This score represents a substantial enhancement of 12.1% over the resample/resize pre-processing method and an improvement of 6.5% over the random crop technique without the incorporation of the proposed embedding. Similarly, the SwinUNETR model attained the highest Dice score of 0.633 when the proposed embedding was utilized. This score surpassed the performance of the resample/resize method by 9.6% and outperformed the random crop approach without the proposed embedding by 7.6%.

**Table 1:** Performance comparison of UNETR and SwinUNETR model with different pre-processing method with and without proposed positional embedding (PPE).

Model	Pre-processing Method	Embedding	Mean Dice Score	HD95 (mm)
UNETR	Resample	Regular	0.439	6.53
	Random crop	Regular	0.495	4.74
	Random crop	Regular + *PPE*	**0.560**	3.35
SwinUNETR	Resample	Regular	0.537	6.90
	Random crop	Regular	0.557	4.59
	Random crop	Regular + *PPE*	0.633*	3.04

The HD95 values are computed on true positive components as infarct segmentation has multiple region targets. With proposed embedding and random cropping, the performance improvement is significant.

*Wilcoxon signed-rank test p-value of the best PPE method compared to without PPE methods after Bonferroni correction < 0.0001.

In Table 2, we compare the performance of our proposed method against two popular CNN based model UNET^16,17^ and DynUNET^18,19^ for the task of infarct segmentation. The UNETR model with the proposed embedding outperformed the UNET model, although it fell short of matching the Dice score achieved by the DynUNET model by 1.0%. However, SwinUNETR model with proposed embedding exhibited superior performance, surpassing the DynUNET model by 6.3% and the UNET model by 11.2% in terms of Dice score.

**Table 2:** Performance comparison of transformer based UNETR and SwinUNETR models with and without the proposed embedding with CNN architectures.

Model	Mean dice score	HD95
UNETR	0.495	4.74
SwinUNETR	0.557	4.59
UNET	0.521	6.07
DynUNET	0.570	5.52
UNETR + *PPE*	0.560	3.35
SwinUNETR + *PPE*	0.633	3.04

Table 3 presents the results of the Wilcoxon signed-rank test comparing the p-values of our proposed embedding method against alternative approaches for both the UNETR and SwinUNETR architectures. Notably, the p-value was found to be <0.0001 for the proposed embedding compared to all other methods in both architectures, indicating a statistically significant improvement.

**Figure 3:**
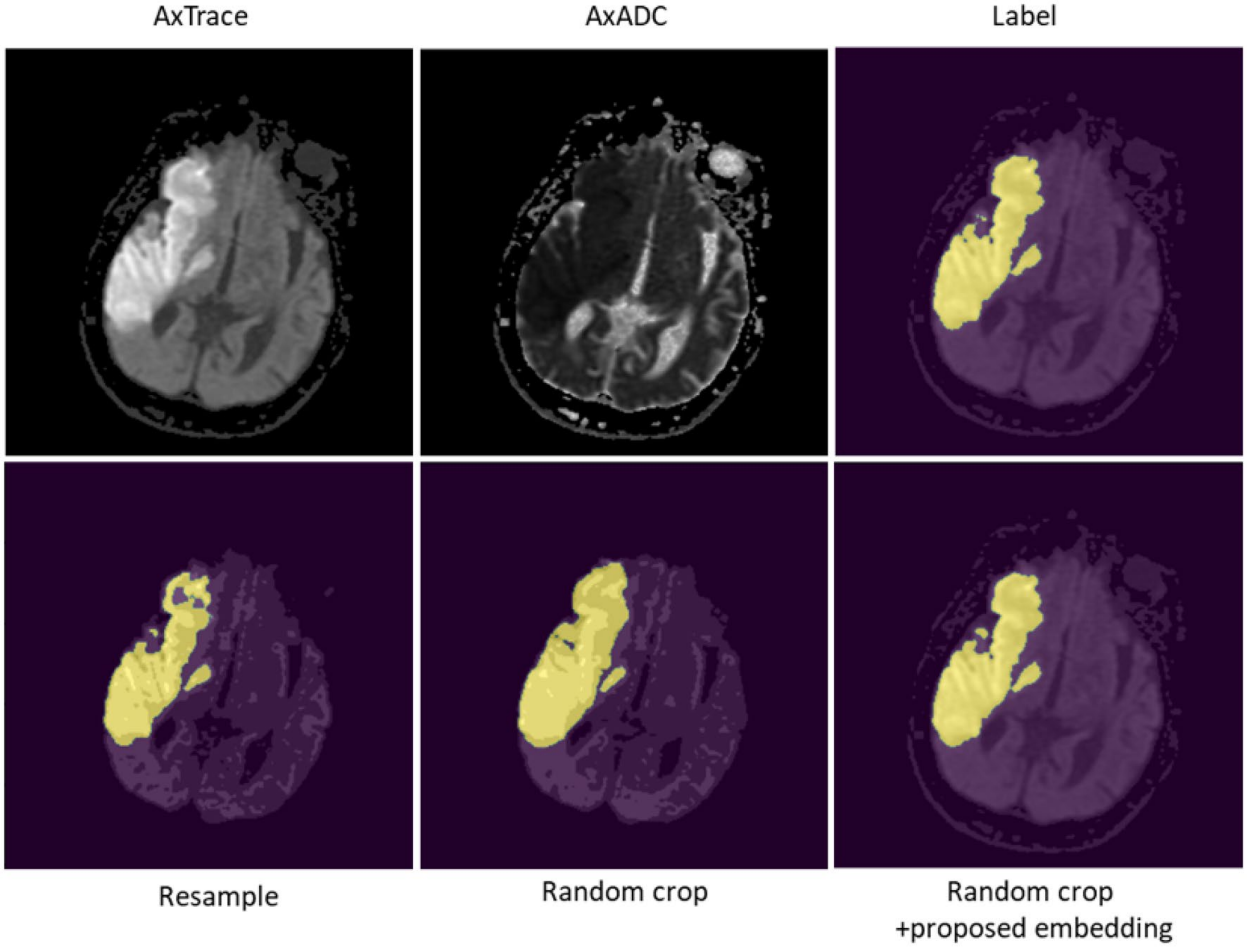
Qualitative comparison of different methods with and without proposed positional embedding. An illustrative segmentation example of the predicted labels which demonstrate differences in methods. First row consists of single slice of AxTrace, AxADC and corresponding infarct ground truth. Second row consists of segmentation output of different methods.

**Figure 4:**
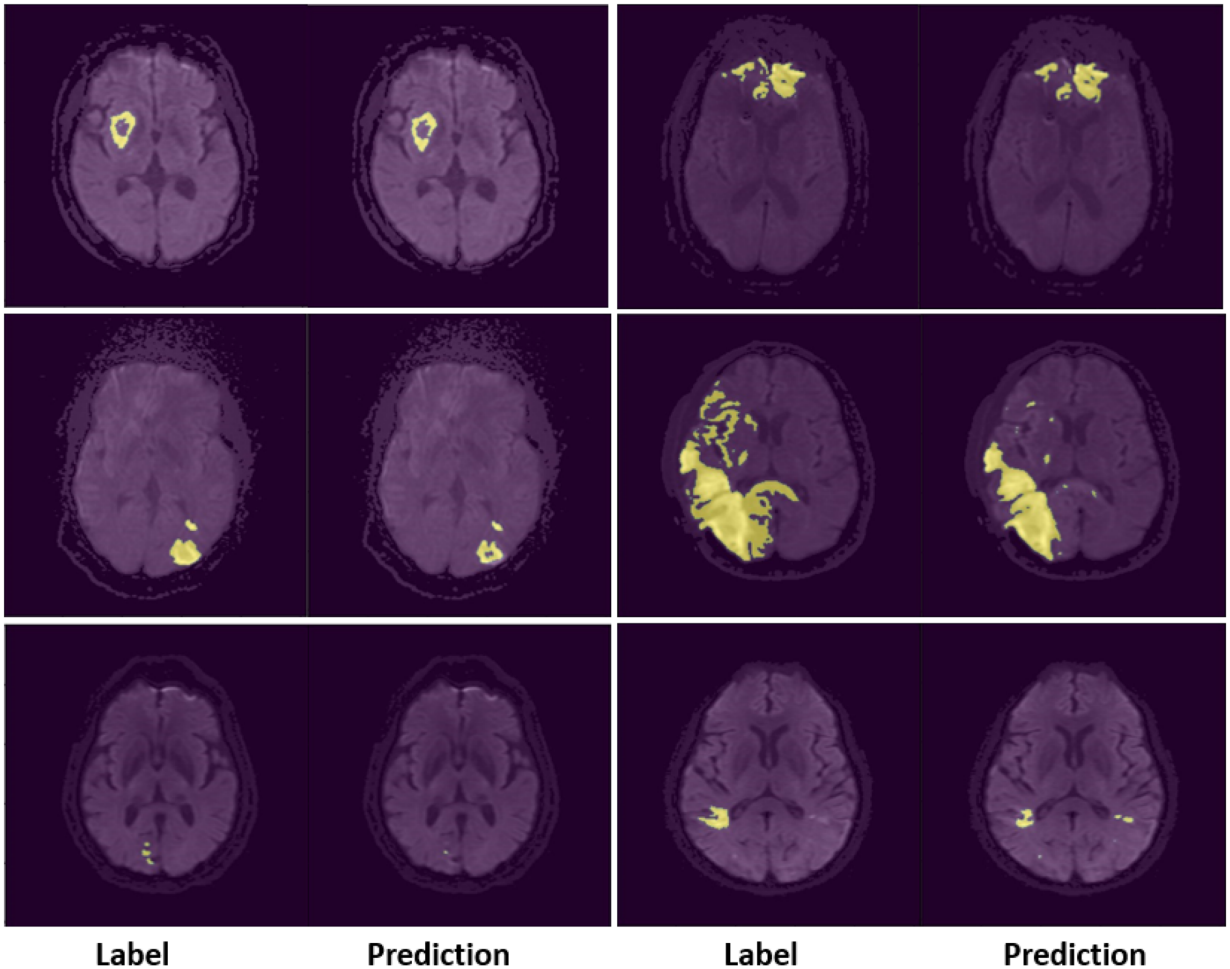
A typical segmentation example of the predicted labels using proposed co-ordinate based embedding. The first row depicts around 75th percentile performance of two samples based on the Dice score. Second and third rows depict around 50 percentile and around 25 percentile performance respectively.

Qualitative comparisons of acute/subacute infarct segmentations of different methods are presented in Fig. 3. The segmentation from the model with resampling exhibits holes in the anterior part of the infarct. The segmentation from the model with random crop does not exhibit the holes but over-segments the infarct. The segmentation from the proposed model demonstrates superior performance in capturing the intricate details of the infarct region, highlighting its ability to accurately delineate the fine-grained features compared to the other models. In addition, the segmentation outputs of SwinUNETR for several cases in the test dataset are illustrated in Fig. 4.

**Table 3:** Wilcoxon signed-rank test p-value comparison of different methods after Bonferroni correction.

Model	Hypothesis	P-value
UNETR	Random Crop+*PPE* Vs Resample	<0.0001
	Random Crop+*PPE* Vs Random Crop	<0.0001
SwinUNETR	Random Crop+*PPE* Vs Resample	<0.0001
	Random Crop+*PPE* Vs Random Crop	<0.0001

In Table 4 we evaluated the effectiveness of the angular frequency of the sinusoidal structure. The performance difference was very minimal for two different angular frequencies. The Wilcoxon signed-rank test p-value of angular frequency 1 compared to angular frequency 0.1 was 0.5021. In Table 5 we evaluated the effectiveness of the scaling factor of the PPE on segmentation performance using the SwinUNETR model. Our analysis revealed that with a scaling factor of 100, the model attained its highest mean dice score of 0.633. However, a decrease in the scaling factor to 10 and 1 resulted in a noticeable decline in performance.

**Table 4:** Effect of different angular frequency($\omega$) on infarct segmentation performance using SwinUNETR.

Angular frequency	Mean Dice Score
1	0.633
0.1	0.628

**Table 5:** Effect of different scaling factor(S) on infarct segmentation performance using SwinUNETR.

Scaling factor	Mean dice score
1	0.569
10	0.602
100	0.633
1000	0.629
10000	0.626

The BraTS 2021 validation dataset findings obtained with the UNETR and SwinUNETR models are displayed in Table 6. In this case, there was no significant improvement in performance with the proposed embedding.

**Table 6:** Performance comparison on BraTS 2021 validation dataset with UNETR and SwinUNETR models.

Model	Dice TC	Dice WT	Dice ET	Mean dice score
UNETR	0.837	0.825	0.839	0.833
UNETR + *PPE*	0.841	0.834	0.853	0.843
SwinUNETR	0.874	0.872	0.862	0.869
SwinUNETR + *PPE*	0.865	0.894	0.858	0.872

The effect of the proposed embedding with and without the regular positional embedding of the Transformer is presented in Table 7. We observed that to fully leverage the capabilities of the Transformer, it was essential to incorporate regular positional embedding alongside PPE. Without the regular positional embedding the performance of SwinUNETR model dropped by 2.2% and the mean dice score of infarct segmentation was 0.611 compared to 0.633 with both regular and the proposed embedding.

**Table 7:** Effect of PPE with and without regular embedding on infarct segmentation performance using SwinUNETR.

Embedding	Mean dice score
*PPE*	0.611
Regular + *PPE*	0.633

## Discussion

We proposed a co-ordinate-based pixel-wise positional embedding method for medical image data. This embedding provides physical co-ordinate and resolution information to the Transformer and works with existing Transformer pipelines. It can be simply added to the input images in Transformer-based architectures without any change in the models. Our embedding demonstrated significant enhancement of the performance of two commonly used Transformer based models - UNETR and SwinUNETR - for infarct segmentation.

In Transformer-based models, positional embedding typically embeds only the position of each patch relative to the source image, losing the relationship to physical co-ordinates. In medical imaging, spatial relationships are captured and inherently preserved across contrasts and across imaging sessions. Our proposed co-ordinate based positional embedding enables our model to use this information to improve its performance. We have achieved 6.5% and 7.6% performance improvement in UNETR and SwinUNETR model respectively by using both patch-wise and co-ordinate based positional embedding. By combining these two, we can provide both information of patches, which is essential for self-attention, as well as co-ordinate information of each subject, which can help model to understand field of view, resolution, and size difference of different inputs. While doing so, we can also eliminate resampling/resizing in our pre-processing steps.

We have achieved significant improvement in infarct segmentation. The accurate identification and localization of recent brain ischemic infarcts is a critical element in therapy decisions for patients with stroke symptoms and has an impact on outcomes^20^. By improving the accuracy of automated brain ischemic infarct segmentation, we hope to contribute to improving patient outcomes.

It is important to take into account a few limitations while interpreting our results. The annotations were done by one radiologist and may not reflect observer variability. Additionally, the data coming primarily from academic centers whose demographics may not be representative. Our experiments demonstrated improvements when image geometry varied between imaging studies but was the same between images within an imaging study. However, this methodology can also encode information from multiple images with different geometry within the same imaging study which is common in multiparametric medical images. In future, we want to use different size images for different contrasts for the same patient’s imaging study. Furthermore, This methodology exhibits suitability primarily for larger datasets, as the Transformer model necessitates a substantial volume of data to attain convergence. Also, another limitation is that we did not analyze different subgroups in this study. This is something important to consider for future research, but it is beyond the scope of this current work.

We also observed, the addition of proposed embedding on BraTS 2021 dataset could not provide the additional boost to the performance. This may be because of every data sample of BraTS dataset has same origin, size and resolution and the embedding will be same for all the samples. Thus the proposed embedding is more useful if the dataset is heterogeneous.

## Conclusion

In this paper, we introduced a unique co-ordinate-based pixel wise positional embedding for medical images which is created from physical co-ordinate and resolution information included in metadata. We validated the effectiveness of the proposed embedding with UNETR and SwinUNETR model and it enhanced the performance of these models significantly. In conclusion, the proposed embedding has shown potential to help Transformer understanding different field of view, resolution, and matrix size of medical images. This method could be the foundation for new positional embedding techniques for Transformer based models in medical image analysis.

### Supplementary Information

Supplementary Information.

## Data Availability

The training, validation, and test datasets used for this study are protected patient information. Some data may be available for research purposes from the corresponding author upon reasonable request.

## Code availability

The code base for the deep-learning framework makes use of proprietary components and we are unable to publicly release the code base. However, all experiments and implementation details are described in sufficient detail in the Methods to enable independent replication with non-proprietary libraries.
