# Supplementary material for: Co-ordinate-based positional embedding that captures resolution to enhance transformer’s performance in medical image analysis
Source: Sci Rep. 2024 Apr 23;14:9380. doi: 10.1038/s41598-024-59813-x (PMC11039716; doi:10.1038/s41598-024-59813-x)
Supplement: Supplementary file 1 — Supplementary Information. [file 41598_2024_59813_MOESM1_ESM.docx]

| Dataset | Manufacturer | Model Name | Number of cases | Number of Slices | Slice Thickness | Echo Time | Repetition Time |
| --- | --- | --- | --- | --- | --- | --- | --- |
| Training | Siemens | Symphony, Aera, Skyra, Avanto, Amira, Verio, Biograph_mMR | 92 | 19-34 | 4.0-6.0 | 66-100 | 3200-9100 |
|  | GE | Genesis Signa, Optima MR450w, Discovery MR750, Signa HDxt | 1050 | 18-66 | 2.5-6.0 | 69.8-131.8 | 4000-17000 |
| Validation | Siemens | Symphony, Aera, Skyra, Amira, Verio | 11 | 19-33 | 4.0-5.0 | 66-98 | 3200-8600 |
|  | GE | Optima MR450w, Signa HDxt, Discovery MR750 | 122 | 28-76 | 2.5-5.0 | 77.6-127.3 | 4000-17000 |
| Test | Siemens | Symphony, Skyra, Amira | 6 | 19-34 | 5.0 | 94-98 | 3200-4900 |
|  | GE | Optima MR450w, Genesis Signa, Discovery MR750, Signa HDxt | 137 | 18-62 | 2.5-6 | 77.9-122.6 | 4000-17000 |

**Table:** Training, validation and test dataset details with manufacturer name, model, number of cases, number of slices, slice thickness, echo time and repetition time.


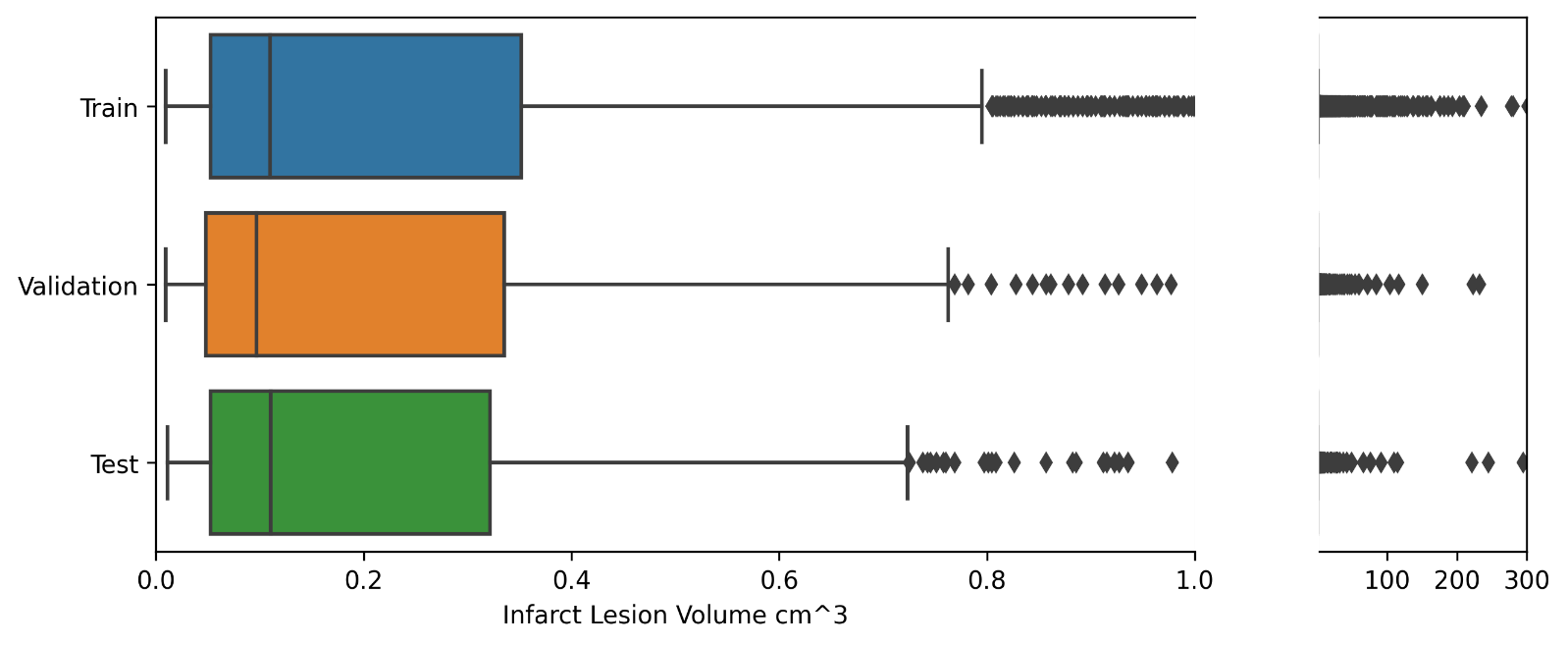


**Figure**: Infarct lesion distribution for train, validation, and test sets. The median values are 0.1099 cm^3^, 0.0923 cm^3^ and 0.1103 cm^3^ respectively for train, validation, and test sets. Please note that we break the plot into two parts. The left one is to show the location of the box where the middle 50% of all lesions are present. The range of the outliers are shown in the right side with a compressed scale between 1.0 cm^3^ and 300 cm^3.^
